# Association of hyperuricemia and gamma glutamyl transferase as a marker of metabolic risk in alcohol use disorder

**DOI:** 10.1038/s41598-020-77013-1

**Published:** 2020-11-18

**Authors:** Anna Hernández-Rubio, Arantza Sanvisens, Ferran Bolao, Clara Pérez-Mañá, Nuria García-Marchena, Carla Fernández-Prendes, Alvaro Muñoz, Roberto Muga

**Affiliations:** 1 Department of Internal Medicine, Hospital Universitari Germans Trias i Pujol, 08916 Badalona, Spain; 2 Department of Medicine, Universitat Autònoma de Barcelona, 08035 Barcelona, Spain; 3 Department of Internal Medicine, Hospital Universitari de Bellvitge-IDIBELL, Universitat de Barcelona, L’Hospitalet de Llobregat, Barcelona, Spain; 4 Department of Clinical Pharmacology, Hospital Universitari Germans Trias i Pujol, Universitat Autònoma de Barcelona, Badalona, Spain; 5 Department of Clinical Analysis and Biochemistry, Laboratori Clinic Metropolitana Nord, Hospital Universitari Germans Trias i Pujol, Universitat Autònoma de Barcelona, Badalona, Spain; 6 Department of Epidemiology, Johns Hopkins University, Bloomberg School of Public Health, Baltimore, MD USA

**Keywords:** Metabolic disorders, Addiction

## Abstract

Excessive alcohol consumption leads to overproduction of urates and renal function plays a critical role in serum uric acid levels. We aimed to assess associations of hyperuricemia in patients with alcohol use disorder (AUD) and comparable Glomerular Filtration Rate (GFR). A total of 686 patients undergoing treatment for AUD between 2013 and 2017 were eligible (77% men); age at admission was 47 years [interquartile range (IQR), 40–53 years], age of onset of alcohol consumption was 16 years [IQR, 16–18 years] and the amount of alcohol consumed was 160 g/day [IQR, 120–240 g/day]. Body Mass Index was 24.7 kg/m^2^ [IQR, 21.9–28.4 kg/m^2^], eGFR was 105 mL/min/1.73 m^2^ [IQR, 95.7–113.0 mL], 9.7% had metabolic syndrome and 23% had advanced liver fibrosis (FIB-4 > 3.25). Prevalence of hyperuricemia was 12.5%. The eGFR-adjusted multivariate analysis showed that relative to patients with GGT ≤ 50, those with GGT between 51 and 300 U/L and those with GGT > 300 U/L were 4.31 (95% CI 1.62–11.46) and 10.3 (95% CI 3.50–29.90) times more likely to have hyperuricemia, respectively. Our data shows that hyperuricemia in the context of AUD is strongly associated with serum GGT levels and suggest an increased cardio-metabolic risk in this population.

## Introduction

Uric acid is a heterocyclic organic compound synthesized primarily in the liver and small intestine and the final product of purine catabolism. Other than purines obtained from food products, the primary endogenous source of urates is nucleic acids, specially adenine and guanine^1^. The kidney is responsible for removing the majority (> 70%) of urates produced daily^2^. Excessive alcohol consumption, obesity, metabolic syndrome (MetS) and food products with a high concentration of purines or fructose have been associated with hyperuricemia^3^.

The estimated prevalence of hyperuricemia in the general population is around 5% and it seems to be much higher in hospitalized patients^4,5^. There are no universally accepted cut-off levels of serum uric acid (SUA) although serum concentrations from 5.0 to 7.5 mg/dL, depending on sex, are considered high. More than two-thirds of patients with hyperuricemia remain clinically asymptomatic, although the risk of developing gout is directly related to SUA levels^3,6^.

The mechanisms explaining the pathophysiology of hyperuricemia includes the excessive urate production and the reduced renal and extra-renal excretion. The under-excretion of urates is the most frequent etiology (90% of cases) of hyperuricemia and it may be classified into primary conditions characterized by a decrease in the tubular secretion of urates, or into secondary conditions due to renal failure or other causes such as diabetes, alcoholic ketosis and lactic acidosis^2,7^.

With respect to the role of ethanol in the SUA levels, excessive alcohol consumption accelerates adenosine triphosphate degradation in the liver and increases lactic acid levels in blood during the degradation of ethanol. Ethanol also increases the plasma concentration and decreases the urinary excretion of purine bases (i.e. hypoxanthine and xanthine) via the acceleration of adenine nucleotide degradation and the possible inhibition of the xanthine dehydrogenase activity. In addition, chronic alcohol consumption might diminish the urinary excretion of uric acid due to dehydration and ketoacidosis^8,9^.

Hyperuricemia has been associated with an increased risk of cardiometabolic and renal disease^10,11^. In addition, hyperuricemia may increase the risk of hepatic steatosis and gluconeogenesis, which contribute to MetS^12^. In parallel, patients with alcohol use disorder (AUD) may have a higher risk of cardiovascular risk factors and it is estimated that up to 20% of individuals with excessive alcohol consumption have MetS^13,14^.

Studies involving moderate alcohol drinkers have shown that SUA levels increase proportionally to the amount of alcohol consumed and that this increase is more pronounced among individuals drinking beer or distillates^15^. However, associations of alcoholic beverages and SUA may be due to the effect of non-alcoholic components of beverages (i.e. purine content of beer, antioxidants in wine); these components of alcoholic beverages may play a role in the variation of SUA levels^15,16^.

It should be noted that the majority of studies on SUA and alcohol consumption have focused on hospitalized patients with acute, concurrent illness (i.e. bacterial pneumonia) or in the general population^4,15,17–19^. However, few studies have taken into account the role of GFR and impaired renal function in SUA levels which may affect prevalence and associations of hyperuricemia. The kidney has been recognized as a main regulator of SUA and renal urate excretion is determined by the balance of the reabsortion and secretion of urates^2^.

We hypothesize that the risk of hyperuricemia in the context of excessive alcohol consumption is multifactorial and related to multiple health outcomes which may have implications for clinical practice. We aimed to characterize associations of hyperuricemia in AUD patients with comparable GFR.

## Methods

This was a cross-sectional study in AUD patients consecutively admitted for detoxification in two hospital units in metropolitan Barcelona, Spain: Hospital Universitari de Bellvitge in L’Hospitalet de Llobregat and Hospital Universitari Germans Trias i Pujol in Badalona. All patients were consecutively admitted between January 2013 and November 2017; patients were referred to the Addiction Unit by primary care physicians and specialists in addiction medicine at outpatient clinics.

All patients received a diagnosis of alcohol use disorder according to the Diagnostic and Statistical Manual of Mental Disorders, 5th Ed (DSM-5)^20^. Information was collected on socio-demographic variables (sex, age, education level and employment). On admission day, patients underwent an interview including questions on alcohol consumption such as daily quantity and type of alcohol (beer, wine, liquor/distilled spirits) and age at onset of alcohol use. Alcohol consumption was quantified in grams of ethanol per day. Current use of other substances (i.e., cocaine) was ascertained by urine detection at admission. Anthropometric data (height and weight) were also obtained.

Peripheral blood samples for determining biochemical and hematological parameters were collected the second day of admission after overnight fasting. Blood was collected in heparin plasma tubes and transferred to the laboratories. Hematological parameters (i.e., hemoglobin, mean corpuscular volume (MCV), platelet count, leucocytes, and lymphocytes) and erythrocyte sedimentation rate (ESR) were measured using automated hematological profile analyzers; biochemical parameters [i.e., glucose, urate, albumin, total cholesterol, triglycerides, urea, creatinine, aspartate aminotransferase (AST), UA, alanine aminotransferase (ALT), and gamma-glutamyl transferase (GGT)] were measured with standard enzymatic methods using multichannel automatic analyzers; specifically, the enzymatic colorimetric method was used to assess the SUA level. Overall inter- and intra-assay variability was 3.6–12.4%. The clinical chemistry and hematology laboratories of both hospitals complied with UNE-EN-ISO9001:2015 standards which accredited them in their respective areas.

Pharmacological treatment during admission included benzodiazepines, vitamin B complex, and other pharmacotherapy depending on medical co-morbidities, and severity of alcohol withdrawal.

On average, the length of stay was 7 days, and at discharge, patients were advised to attend follow-up visits at the outpatient clinic. Other details regarding admission for the treatment of AUD can be found elsewhere^21,22^.

A total of 739 patients were admitted during the study period but patients treated with diuretics (n = 36), uricosuric agents (n = 12), or both (n = 5) were excluded yielding 686 for analysis presented here. The established cut-off value for hyperuricemia was > 7.2 mg/dL in men and > 6 mg/dL in women based on the reference values provided by the hospital laboratories.

Renal function was assessed by the estimated glomerular filtration rate (eGFR), calculated using creatinine, age, sex and ethnicity through the Chronic Kidney Disease Epidemiology Collaboration equation (CKD-EPI).

Patients were evaluated for liver fibrosis using the FIB-4 index, according to the following equation:

$$ FIB4 = \frac{Age\;[years] \times AST\;[U/L]}{{Platelet\;[10^{9} /L] \times \sqrt {ALT\;[U/L]} }} $$

and FIB-4 values > 3.25 indicated advanced liver fibrosis^23^.

MetS was defined as Body Mass Index (BMI) > 25 kg/m^2^ and/or age > 40 years and two or more of the following conditions: serum glucose > 110 mg/dL, triglycerides > 150 mg/dL, HDL-cholesterol < 40 mg/dL in men and < 50 mg/dL in women, and blood pressure > 130/85 mmHg or pharmacologic treatment for hypertension^24^.

All patients gave written informed consent, and the study design was approved by the Ethics Committee of the Hospital Universitari Germans Trias i Pujol (approval number CEXT042013). The methods complied with the ethical standards for medical research and the principles of good clinical practice in accordance with the World Medical Association’s Declaration of Helsinki.

### Statistical analysis

Descriptive statistics were expressed as median (interquartile range (IQR)) for quantitative variables and as absolute frequencies and percentages for qualitative variables.

We used the Chi-square test for qualitative variables and Student’s *t* test or Mann–Whitney *U* test in quantitative variables to analyze differences in patients with and without hyperuricemia. Correlations were determined using Pearson’s correlation coefficient.

Logistic regression models were applied to establish factors associated with hyperuricemia. Sociodemographic variables (age and sex), ESR, albumin and GGT levels, FIB-4, and MetS were analyzed after adjusting for eGFR; for these analysis the eGFR was considered a continuous variable and GGT levels were stratified as ≤ 50 U/L, 51–300 U/L, > 300 U/L, altered ESR was defined as > 20 mm and hypoalbuminaemia < 35 g/L.

The covariates included in the multivariate analysis were those that were found to be statistically significant in the eGFR-adjusted univariate analysis.

Sensitivity analyses of logistic regression models were performed to further analyze associations of hyperuricemia when using different SUA cut-offs. Lower cut-off values (6.0 mg/dL for men and 5.0 mg/dL for women), higher cut-off values (8.0 mg/dL for men and 6.5 mg/dL for women), and a single cut-off point (7.0 mg/dL for both sexes) were used.

*P* values < 0.05 were considered statistically significant. Statistical analyses were performed using Stata software (version 11.1, College Station, Texas, USA).

## Results

A total of 686 patients (77% men) were eligible, with a median age of 47 years [interquartile range (IQR), 40–53 years]. The age of onset of alcohol consumption was 16 years [IQR, 16–18 years] and the amount of alcohol consumed was 160 g/day [IQR, 120–240 g/day]. BMI was 24.7 kg/m^2^ [IQR, 21.9–28.4 kg/m^2^], eGFR was 105 mL/min/1.73 m^2^ [IQR, 95.7–113.0 mL], 9.7% had MetS and 23% had advanced liver fibrosis (FIB-4 > 3.25). The prevalence of hyperuricemia was 12.5%.

Sociodemographic characteristics and data on the consumption of alcohol and other substances in patients with and without hyperuricemia are shown in Table 1. Patients presenting with hyperuricemia were older than those without hyperuricemia (mean 48.6 vs. 46.4 years, *P* = 0.041). Sex, educational level, employment and characteristics related to alcohol consumption and other substances were similar in those with and without hyperuricemia.

**Table 1:** Characteristics of patients with and without hyperuricemia admitted for the treatment of AUD in metropolitan Barcelona, Spain.

	Patients with hyperuricemia* N = 86	Patients without hyperuricemia N = 600	*P* value
**Sociodemographic**			
Women, n (%)	20 (23.3)	136 (22.7)	0.903
Age at admission, median (IQR)	48 (41–54)	46 (40–53)	0.095
Employment			
Non-missing	78	463	0.033
Unemployed, n (%)	30 (38.5)	228 (49.2)	
Employed, n (%)	22 (28.2)	141 (30.4)	
Permanent disability/pensioner, n (%)	26 (33.3)	94 (20.3)	
Completed educational level			
Non-missing	74	507	0.109
Illiterate, n (%)	4 (5.4)	18 (3.5)	
Primary, n (%)	14 (18.9)	164 (32.3)	
Secondary, n (%)	45 (60.8)	271 (53.4)	
University, n (%)	11 (14.9)	54 (10.6)	
**Alcohol and other substances**			
Age of onset alcohol consumption			
Non-missing	79	600	0.268
Median (IQR)	16 (16–18)	16 (16–18)	
Antecedent of AUD treatment			
Non-missing	85	543	0.998
n (%)	62 (72.9)	396 (72.9)	
Amount of alcohol consumption (g/day)			
Non-missing	81	558	0.477
Median (IQR)	190 (110–250)	160 (120–240)	
Current cocaine use			
Non-missing	77	544	0.409
n (%)	9 (11.7)	83 (15.3)	
Current opiate use			
Non-missing	77	540	0.490
n (%)	1 (1.3)	14 (2.6)	
Type of alcoholic beverage			
Non-missing	68	426	0.958
Only wine, n (%)	6 (8.8)	45 (10.6)	
Only beer, n (%)	25 (36.8)	145 (34.0)	
Only liquor and/or distilled, n (%)	7 (10.3)	45 (10.6)	
≥ two types of alcohol, n (%)	30 (44.1)	191 (44.8)	

*AUD* alcohol use disorder.

*SUA > 7.2 mg/dL in men and > 6 mg/dL in women.

The results of laboratory parameters upon admission are shown in Table 2. Patients with hyperuricemia showed higher prevalence of elevated ESR (*P* = 0.010), elevated GGT (*P* < 0.001), and lower eGFR (*P* = 0.001) than those without hyperuricemia. Specifically, the probability of presenting with hyperuricemia increased 1.5-fold for each decrease of 10 mL/min/1.73 m^2^ in the eGFR. A total of 24 (30.8%) patients among those with hyperuricemia and 121 (22%) among those without hyperuricemia had advanced liver fibrosis (FIB-4 > 3.25) (*P* = 0.087).

**Table 2:** BMI and laboratory parameters in AUD patients with and without hyperuricemia.

	Patients with hyperuricemia* N = 86	Patients without hyperuricemia N = 600	*P* value
**Antropomethric**			
BMI (kg/m^2^)			
Non-missing	81	574	< 0.001
Median (IQR)	28.7 (25.1–32.7)	24.2 (21.8–32.7)	
**Haematology**			
Platelet count (× 10^9^ L)			
Non-missing	86	596	0.045
Median (IQR)	185.5 (142–236)	206.0 (149–253)	
Haemoglobin (g/dL)			
Non-missing	86	597	0.020
Median (IQR)	13.8 (12.5–14.9)	14.3 (13.3–15.4)	
MCV (fl)			
Non-missing	86	591	0.893
Median (IQR)	96 (92–100)	96 (92–100.5)	
ESR (mm)			
Non-missing	81	566	0.089
Median (IQR)	10 (5–27)	8 (4–17)	
> 20 mm, n (%)	26 (32.1)	111 (19.6)	0.010
**Biochemistry**			
Glucose (mg/dL)			
Non-missing	86	587	0.031
Median (IQR)	95.7 (86.5–106)	90.1 (84.7–99.1)	
Total cholesterol (mg/dL)			
Non-missing	86	597	0.188
Median (IQR)	202 (161–241)	194 (163–224)	
Triglycerides (mg/dL)			
Non-missing	86	598	< 0.001
Median (IQR)	152 (99–247)	109 (78–159)	
Albumin (g/L)			
Non-missing	86	588	0.795
Median (IQR)	40 (36–43)	39.8 (37–42)	
< 35 g/L, n (%)	19 (22.1)	85 (14.5)	0.067
eGFR (mL/min/1.73 m^2^)			
Non-missing	86	595	< 0.001
Median (IQR)	97.9 (85.6–108.5)	106.2 (96.6–113.4)	
AST (U/L)			
Non-missing	78	552	0.010
Median (IQR)	46.9 (30–88.2)	36.3 (21–71.7)	
ALT (U/L)			
Non-missing	86	595	0.007
Median (IQR)	40.6 (22.8–66)	31 (17.4–55)	
GGT (U/L)			
Non-missing	85	592	< 0.001
Median (IQR)	211.8 (102–702.6)	102 (42–286)	
≤ 50, n (%)	8 (9.4)	172 (29.0)	< 0.001
51–300, n (%)	42 (49.4)	279 (47.1)	
> 300, n (%)	35 (41.2)	141 (23.8)	
SUA (mg/dL), Median (IQ R)	7.8 (7.4–8.5)	4.9 (4.1–5.7)	< 0.001
**Comorbidity**			
Advanced liver fibrosis, FIB-4 > 3.25			
Non-missing	78	549	0.087
n (%)	24 (30.8)	121 (22.0)	
MetS			
Non-missing	81	570	0.204
n (%)	11 (13.6)	52 (9.1)	

*BMI* Body Mass Index, *MCV* mean corpuscular volume, *ESR* erythrocyte sedimentation rate, *AST* aspartate aminotransferase, *ALT* alanine aminotransferase, *GGT* gammaglutamyl transferase, *SUA* serum uric acid, *MetS* metabolic syndrome.

*SUA > 7.2 mg/dL in men and > 6 mg/dL in women.

**Figure 1:**
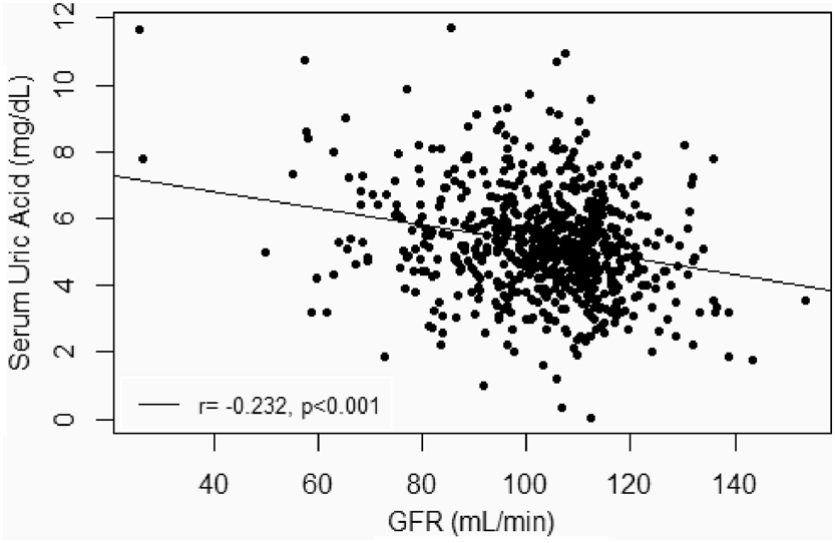
Correlation of serum uric acid and eGFR in 686 patients admitted for the treatment of AUD in metropolitan Barcelona, Spain. *GFR* glomerular filtration rate.

Figure 1 shows the negative correlation between SUA and eGFR. Specifically, for each decrease of 10 mL/min/1.73 m^2^ in the eGFR, SUA increased by 0.24 mg/dL (r = − 0.232, *P* < 0.001).

The eGFR-adjusted, logistic regression models are shown in Table 3. Elevated ESR, increased GGT levels, and advanced liver fibrosis were significant predictors of hyperuricemia in the eGFR-adjusted univariate analysis. Multivariate analysis showed that serum GGT was the only independent predictor of hyperuricemia (*P* < 0.001). Specifically, relative to patients with GGT ≤ 50, those with GGT between 51 and 300 and those with GGT > 300 U/L were 4.31 (95% CI 1.62–11.46) and 10.3 (95% CI 3.50–29.90) times more likely to have hiperuricemia, respectively. The drastic differences between the odds ratios associated with FIB-4 obtained from univariate and multivariate logistic regression are due to strong confounding with GGT. Specifically, among those with FIB-4 ≤ 3.25, 31.9%, 53.2% and 14.9% had GGT ≤ 50, 51 < GGT ≤ 300 and GGT > 300 U/L, respectively; and in contrast, among those with FIB-4 > 3.25, the great majority had GGT > 300 with 2.1%, 30.6% and 67.4% having GGT ≤ 50, 51 < GGT ≤ 300 and GGT > 300 U/L, respectively. Likewise, among those with ESR ≤ 20 mm, 28.7%, 49.4% and 21.9% had GGT ≤ 50, 51 < GGT ≤ 300 and GGT > 300 U/L, respectively; and in contrast, among those with ESR > 20 mm, 17.2%, 43.3% and 39.5% had GGT ≤ 50, 51 < GGT ≤ 300 and GGT > 300 U/L, respectively.

**Table 3:** Logistic regression model for associations of hyperuricemia in patients undergoing treatment for AUD.

	aOR* (95%CI)	*P* value	Multivariate aOR* (95%CI)	*P* value
Women	0.79 (0.45–1.40)	0.422	–	
Age	1.00 (0.98–1.03)	0.805	–	
ESR > 20 mm	1.90 (1.12–3.23)	0.019	1.34 (0.73–2.47)	0.344
Albumin < 35 g/L	1.74 (0.97–3.11)	0.064	–	
GGT (U/L)				
≤ 50	1		1	
51–300	3.56 (1.60–7.90)	0.002	4.31 (1.62–11.46)	0.003
> 300	8.14 (3.51–18.9)	< 0.001	10.27 (3.53–29.90)	< 0.001
MetS	1.31 (0.64–2.70)	0.462	–	
Advanced liver fibrosis, FIB-4 > 3.25	2.00 (1.15–3.46)	0.013	0.99 (0.51–1.92)	0.982

*ESR* erythrocyte sedimentation rate, *GGT* gamma-glutamyl transferase, *MetS* metabolic syndrome.

*Glomerular filtration rate-adjusted.

The results of sensitivity analyses using different cut-off values for SUA were consistent with those presented here.

## Discussion

The role of excessive alcohol consumption in the risk of hyperuricemia/gout and metabolic complications is well known. However, few studies have analyzed the determinants of hyperuricemia in patients with eGFR-adjusted (i.e., comparable renal function). The kidney is a key regulator of circulating uric acid levels by reabsorbing 90% of filtered urate and results from this large case-series indicates that one in eight patients with AUD had hyperuricemia. In addition, prevalence would reach 14% if we had included patients who were treated with uricosuric agents or diuretics upon admission; such similar prevalence of hyperuricemia has been reported in individuals with heavy alcohol consumption^19,25^. More important, our data indicates a strong dose–response relationship between serum GGT and SUA levels after adjusting for potential confounders (i.e., increased GGT serum levels independently contributed to increases in SUA levels) and this finding may have implications in clinical practice and public health interventions. Studies have shown the role of serum GGT as a surrogate of oxidative stress^26–28^ and pointed out that both SUA and GGT are markers of cardio-metabolic risk^28–32^. In this sense, serum GGT is a marker of hepatic intracellular triglyceride accumulation that has been linked to obesity, insulin resistance, atherosclerosis^33,34^, and hyperuricemia regardless of alcohol consumption^35,36^.

Serum GGT may be a contributing factor for hyperuricemia through oxidative stress and insulin resistance. Specifically, serum GGT is associated with hepatic insulin resistance, which in turn is correlated with hyperuricemia due to a reduction in urate excretion^12,37^. On the other hand, GGT plays a critical role in the metabolism of glutathione, a key antioxidant in humans, and there is a correlation between elevated GGT serum levels and oxidative stress leading to steatohepatitis^30,38^. Furthermore, uric acid itself promotes oxidative stress in adipocytes by increasing monocyte chemotactic protein-1 and decreasing adiponectin^39^. In addition, the pro-oxidant action of uric acid is also related to the accumulation of triglycerides in the liver (i.e. steatohepatitis)^12,37^.

Two studies in the general population have analyzed the connection between serum GGT and SUA. One study evaluated normotensive individuals free of liver, kidney, and metabolic diseases and found a positive correlation between serum GGT and urate^28^. The other analyzed the general Japanese population and demonstrated a strong correlation between the two serum markers in both men and women^40^.

To the best of our knowledge, the present study is the first to report serum GGT as an independent contributing factor for hyperuricemia in AUD.

In this study, there were no significant differences in prevalence of hyperuricemia according to the amount and type of alcoholic beverage. A population study based on the US Third National Health and Nutritional Examination Survey (NHANES III) found that SUA levels increased as total alcohol intake increased^15^. With regard to the type of alcoholic beverage, several studies have shown that, in contrast to wine, the consumption of beer and spirits is related to higher SUA levels and to an increased risk of gout^15,41^. Notwithstanding, the absence of significant associations between hyperuricemia and the amount and type of alcohol consumed in our study population might be due to the large amount of alcohol ingested daily. In this respect, few studies have analyzed the association between SUA levels and the drinking pattern in the context of AUD. A population study in Taiwan (2017) involving 11,675 people with alcohol-related diseases, including alcohol dependence, suggested a positive relationship between long-term alcohol dependence and gout risk^17^.

The present study has limitations that should be mentioned. First of all, variables related to the risk factors for hyperuricemia such as dietary habits were not available; in this sense, the link between fructose-rich beverages and purine-rich foods with hyperuricemia/gout is well known^6^. Moreover, some cases of hyperuricemia could be hereditary due to inborn errors of purine and pyrimidine metabolism^42^; in addition, familial and twin studies estimating the heritability of SUA alterations suggest that genetic factors may explain up to 25–60% of the variability in SUA levels^18,43^. Finally, the cross-sectional design of this study did not allow the establishment of causal relationships.

In conclusion, hyperuricemia was found to be relatively common in patients seeking treatment of AUD and closely linked to GGT which suggest an increased cardiometabolic risk in this population.
