# Author Correction: Association of hyperuricemia and gamma glutamyl transferase as a marker of metabolic risk in alcohol use disorder

**DOI:** 10.1038/s41598-023-31311-6

**Published:** 2023-03-14

**Authors:** Anna Hernández‑Rubio, Arantza Sanvisens, Ferran Bolao, Clara Pérez‑Mañá, Nuria García‑Marchena, Carla Fernández‑Prendes, Alvaro Muñoz, Roberto Muga

**Affiliations:** 1 Department of Internal Medicine, Hospital Universitari Germans Trias i Pujol, 08916 Badalona, Spain; 2 Department of Medicine, Universitat Autònoma de Barcelona, 08035 Barcelona, Spain; 3 Department of Internal Medicine, Hospital Universitari de Bellvitge‑IDIBELL, Universitat de Barcelona, L’Hospitalet de Llobregat, Barcelona, Spain; 4 Department of Clinical Pharmacology, Hospital Universitari Germans Trias i Pujol, Universitat Autònoma de Barcelona, Badalona, Spain; 5 Department of Clinical Analysis and Biochemistry, Laboratori Clinic Metropolitana Nord, Hospital Universitari Germans Trias i Pujol, Universitat Autònoma de Barcelona, Badalona, Spain; 6 Department of Epidemiology, Johns Hopkins University, Bloomberg School of Public Health, Baltimore, MD USA

Correction to: *Scientific Reports* 10.1038/s41598-020-77013-1, published online 18 November 2020

In the original version of this Article, Affiliation 1 was incorrectly given as ‘Department of Internal Medicine, Hospital Universitari Germans Trias i Pujol, Universitat Autònoma de Barcelona, 08916 Badalona, Spain’.

The correct affiliations are listed below:

1. Department of Internal Medicine, Hospital Universitari Germans Trias i Pujol, 08916, Badalona, Spain.

2. Department of Medicine, Universitat Autònoma de Barcelona, 08035, Barcelona, Spain.

As a result, the Affiliations have been renumbered.

The original Article has been corrected.

